# Machine learning-based model to predict delirium in patients with advanced cancer treated with palliative care: a multicenter, patient-based registry cohort

**DOI:** 10.1038/s41598-024-61627-w

**Published:** 2024-05-20

**Authors:** Yu Jung Kim, Hayeon Lee, Ho Geol Woo, Si Won Lee, Moonki Hong, Eun Hee Jung, Shin Hye Yoo, Jinseok Lee, Dong Keon Yon, Beodeul Kang

**Affiliations:** 1 Division of Hematology and Medical Oncology, Department of Internal Medicine, Seoul National University Bundang Hospital, Seoul National University College of Medicine, Seongnam, South Korea; 2 Department of Biomedical Engineering, Kyung Hee University, 1732 Deogyeong-daero, Giheung-gu, Yongin, 17104 South Korea; 3 Department of Neurology, Kyung Hee University Medical Center, Kyung Hee University College of Medicine, Seoul, South Korea; 4 Division of Medical Oncology, Department of Internal Medicine, Yonsei Cancer Center, Yonsei University Health System, Seoul, South Korea; 5 Palliative Cancer Center, Yonsei Cancer Center, Yonsei University Health System, Seoul, South Korea; 6 Center for Digital Health, Medical Science Research Institute, Kyung Hee University Medical Center, Kyung Hee University College of Medicine, Seoul, South Korea; 7 Department of Pediatrics, Kyung Hee University College of Medicine, 23 Kyungheedae-ro, Dongdaemun-gu, Seoul, 02447 South Korea; 8 Division of Medical Oncology, Department of Internal Medicine, CHA Bundang Medical Center, CHA University School of Medicine, 59 Yatap-ro, Bundang-gu, Seongnam, 13496 South Korea; 9 Center for Palliative Care and Clinical Ethics, Seoul National University Hospital, Seoul, South Korea

**Keywords:** Cancer, Delirium, Feature importance, Machine learning, Palliative care, Health care, Medical research, Oncology

## Abstract

This study aimed to present a new approach to predict to delirium admitted to the acute palliative care unit. To achieve this, this study employed machine learning model to predict delirium in patients in palliative care and identified the significant features that influenced the model. A multicenter, patient-based registry cohort study in South Korea between January 1, 2019, and December 31, 2020. Delirium was identified by reviewing the medical records based on the criteria of the Diagnostic and Statistical Manual of Mental Disorders, Fifth Edition. The study dataset included 165 patients with delirium among 2314 patients with advanced cancer admitted to the acute palliative care unit. Seven machine learning models, including extreme gradient boosting, adaptive boosting, gradient boosting, light gradient boosting, logistic regression, support vector machine, and random forest, were evaluated to predict delirium in patients with advanced cancer admitted to the acute palliative care unit. An ensemble approach was adopted to determine the optimal model. For k-fold cross-validation, the combination of extreme gradient boosting and random forest provided the best performance, achieving the following accuracy metrics: 68.83% sensitivity, 70.85% specificity, 69.84% balanced accuracy, and 74.55% area under the receiver operating characteristic curve. The performance of the isolated testing dataset was also validated, and the machine learning model was successfully deployed on a public website (http://ai-wm.khu.ac.kr/Delirium/) to provide public access to delirium prediction results in patients with advanced cancer. Furthermore, using feature importance analysis, sex was determined to be the top contributor in predicting delirium, followed by a history of delirium, chemotherapy, smoking status, alcohol consumption, and living with family. Based on a large-scale, multicenter, patient-based registry cohort, a machine learning prediction model for delirium in patients with advanced cancer was developed in South Korea. We believe that this model will assist healthcare providers in treating patients with delirium and advanced cancer.

## Introduction

Delirium, a common neuropsychiatric problem among patients with advanced cancer^1^, can result in extended hospital stays, higher mortality and morbidity rates, increased healthcare costs, and considerable distress for both patients and their family members, as well as healthcare providers^2,3^. Among patients with advanced cancer admitted to the acute palliative care unit (APCU), delirium can affect 42–88% of individuals^4^. However, few comprehensive studies have thoroughly examined its prevalence and potential risk factors^5^. Although effective preventive interventions for delirium in hospital settings are currently lacking, physicians and healthcare providers can alleviate modifiable risk factors within the APCU by providing exercise programs and family support to reduce the occurrence of delirium^6^. Therefore, early recognition and prevention are essential in patients with risk factors for developing delirium^7^.

To date, nurse-administered questionnaires have mainly been used to predict the risk of delirium in hospitalized patients^8^. However, physicians may find it challenging to conduct daily assessments through questionnaires. Machine learning models have recently been introduced^9–14^. Machine learning models were previously used to predict delirium among patients after surgery for degenerative spinal disease^10^, patients admitted to the intensive care unit^11^, hospitalized patients without cognitive impairment^12^, patients admitted to the general ward^13^, and older patients after general surgery^14^. Furthermore, previous study on predicting delirium was also conducted in patients with advanced cancer receiving pharmacological interventions through machine learning models. However, this study was limited to patients taking antipsychotic medications or trazodone, and no operational criteria for determining the precipitating factors of delirium^9^. The area under the receiver operating characteristic curve (AUROC) for these studies ranged from 0.666 to 0.964.

The machine learning model for predicting delirium in patients with advanced cancer has been explored, with suggested advantages^9^. However, this study only considered the decision-tree model, which is largely unstable because a small change in the data can result in a major change in the structure of the model. Therefore, a comprehensive study using machine learning models is needed to more accurately assess the features of delirium in patients with advanced cancer admitted to the APCU. We aimed to develop and compare a variety of machine learning models to predict delirium in patients with advanced cancer admitted to the APCU and investigate the significant features that influenced the machine learning model.

## Materials and methods

### Data source and study population

Our study utilized a multicenter, patient-based registry cohort collected from four hospitals in South Korea: Seoul National University Bundang Hospital, Yonsei University Severance Hospital, CHA University Bundang Medical Center, and Seoul National University Hospital. We identified potential participants as patients with advanced cancer admitted to the APCU at four centers between January 1, 2019, and December 31, 2020. Of the 2328 patients who met the eligibility criteria: (1) aged 20 years or older; (2) diagnosed with advanced solid cancer; and (3) admitted to the APCU. We excluded five patients with a hospital stay exceeding 3 months, six patients transferred to other departments, and three patients with terminal delirium, defined as delirium that occurred within 2 weeks of death. Our final sample consisted of 2314 patients with advanced cancer who were admitted to the APCU and who met all eligibility criteria^15^.

The study protocol received approval from the Institutional Review Boards of each center (CHA University, CHAMC 2021-03-054-002; Seoul National University, H-2103-028-1201; Seoul National University Bundang Hospital, B-2104/681-405; and Yonsei University, 4-2021-0323). The requirement for informed consent was waived by the Institutional Review Board of each center (CHA University; Seoul National University; Seoul National University Bundang Hospital; and Yonsei University) because only anonymized data were examined. The researchers of this study confirm that all methods were performed in accordance with the relevant guidelines and regulations. Especially, this research followed the guidelines outlined in the transparent reporting of a multivariable prediction model for individual prognosis or diagnosis (TRIPOD) statement (Table S1).

### Variables for machine learning

A total of 39 variables were used in this study, and the justification of the selection was selected based on several previous studies predicting delirium and the available variables in the APCU^16–18^. Based on these results, we proceeded with the establishment of a national registry, excluding the use of data for which construction was deemed infeasible. Additionally^19^, within the National Registry Project. The dataset included general information^20,21^ such as age, sex, chemotherapy during hospitalization, living situation, medical aid recipients, education level, use of glasses or hearing aids, and history of alcohol consumption and smoking. Clinical risk factors such as obesity, blood pressure, and body temperature, various laboratory results like blood tests and C-reactive protein levels, and a history of diseases including delirium, cardiovascular disease, diabetes mellitus, respiratory disease, liver disease, mental illness, and head injury were also collected. We aimed to ascertain the onset of delirium in patients with advanced cancer immediately upon APCU admission, hence all baseline datasets consist of data obtained at the time of admission to the APCU.

To identify delirium, we reviewed medical records based on the criteria outlined in the Fifth Edition of the Diagnostic and Statistical Manual of Mental Disorders. A well-trained physician and an academic nurse conducted this detailed review. Based on previous validation study, we did not use the code from the 10th revision of the International Classification of Diseases because it was deemed unreliable with low sensitivity^22^. Instead, we recorded all potential symptoms, signs, and associated medications and had at least two specialists (BDK and YJK) review each case. In case of any disagreement between the specialists, an additional specialist (SHY) was consulted to make the final decision.

The primary objective of this study was to predict the occurrence of delirium in patients with advanced cancer admitted to the APCU using machine learning models. To achieve this, the data were split into a training-to-testing ratio of 80:20, with the training set comprising 1851 (80%) patients and the testing set comprising 463 (20%) patients. Feature normalization was performed by initially computing the mean and standard deviation of each feature within the training set. Subsequently, this normalization procedure was applied to both the training and testing datasets, to ensure that the mean values were centered at zero and the standard deviations were scaled to one. The proposed machine learning models underwent validated through a stratified fivefold cross-validation process on the training data, followed by further validation using independent testing data^23–27^.

### Machine learning models and evaluation metrics

We evaluated seven machine learning algorithms for predicting delirium in patients with advanced cancer: extreme gradient boosting (XGBoost), adaptive boosting (AdaBoost), gradient boosting (GBM), light gradient boosting (LGBM), logistic regression (LR), support vector machine (SVM), and random forest (RF). For these seven machine learning algorithms, which were optimized by input parameters and hyperparameters, we applied an exhaustive search, which used to brute force through all possible combinations of a set of the hyperparameter combination yielding the best performance, with fivefold cross validation for each model to identify the most optimal hyperparameters. To estimate the uncertainty and variability of our results, we calculated the AUROC, sensitivity, specificity, accuracy, and balanced accuracy scores during the fivefold cross-validation process. These metrics were calculated by the following formulas with values of true positive (TP), true negative (TN), false positive (FP), false negative (FN) for binary classification:

$$Sensitivity= TPR=\frac{TP}{TP+FN}$$

$$Specificity=1-FPR=\frac{TN}{TP+FP}$$

$$Balanced\,\, accuracy=\frac{Sensitivity+Specificity}{2}$$

$$AUROC={\int }_{0}^{1}TPR\left({FPR}^{-1}\left(x\right)\right)dx$$

We adopted AUROC, which is commonly used in binary classification and is not sensitive to class imbalances representing the relationship between the true positive rate (TPR) and the false positive rate (FPR) as the threshold changes, as the evaluation metric for measuring the overall performance of the model.

To further enhance the performance of the machine learning model, we employed an ensemble approach. This technique combines multiple models to improve prediction accuracy and robustness. We created various groups of models by combining all possible model combinations and evaluated their performances to determine the best combination. This approach leveraged the strengths of each individual model while mitigating any weaknesses or limitations.

For each of the best performing machine learning models, we investigated the feature importance, which is a measure of how influential a feature was in splitting a class when branching a node in a tree-based model.

We utilized several popular software tools, including Python 3.9.7 (Python Software Foundation, Wilmington, DE, USA), TensorFlow-gpu 2.6.0, Keras 2.6.0, NumPy 1.21.5, Pandas 1.4.1, Matplotlib 3.5.1, and Scikit-learn 1.0.2, to implement the machine learning models^28–30^.

### Machine learning-driven public website development

We also deployed our machine learning model on a public website (http://ai-wm.khu.ac.kr/Delirium/), enabling the prediction of delirium when provided with information from 39 patients. Upon accessing the website, users enter patient information, which is encoded on the website server, allowing for an immediate delirium prediction result. No private information beyond the selected 39 pieces of data needed to be entered, and all entered information was promptly deleted once the prediction result was obtained, ensuring no risk of information exposure.

### Informal consent

The institutional review board of the four centers approved this study and waived the requirement for informed consent because only anonymized data were examined.

### Ethics statement

The protocol was approved by the institutional review boards of the four centers (CHA University, CHAMC 2021-03-054-002; Seoul National University, H-2103-028-1201; Seoul National University Bundang Hospital, B-2104/681-405; and Yonsei University, 4-2021-0323).

## Results

This study was utilized a multicenter patient-based registry cohort collected from four hospitals in South Korea to develop and investigate the machine learning model for predicting delirium in patients with advanced cancer. Table 1 displays the baseline characteristics of the study population. In the original cohort, 165 (7.1%) patients experienced delirium.

**Table 1:** Included variables for an artificial intelligence model and patient information (total n = 2314).

Characteristics	Total (n = 2314)	Delirium group (n = 165)	Non-delirium group (n = 2149)
Age (mean, SD)	66.3 (12.7)	71.6 (10.3)	65.8 (12.8)
Sex (n, %)			
Male	1095 (47.3)	101 (61.2)	994 (46.3)
Female	1219 (52.7)	64 (38.8)	1155 (53.8)
Chemotherapy during hospitalization (n, %)			
No	1660 (71.7)	136 (82.4)	1524 (70.9)
Yes	654 (28.3)	29 (17.6)	625 (29.1)
Living with family (n, %)			
No	1582 (68.4)	118 (71.5)	1464 (68.1)
Yes	732 (31.6)	47 (28.5)	685 (31.9)
Medical aid recipients (n, %)			
No	2210 (95.5)	153 (92.7)	2057 (95.7)
Yes	104 (4.5)	12 (7.3)	92 (4.3)
Education level (n, %)			
High school graduated or under	1095 (47.5)	81 (49.1)	1014 (47.4)
University graduated or higher	1210 (52.5)	84 (50.9)	1126 (52.6)
Visual impairment (wearing glasses) (n, %)			
No	2147 (92.8)	149 (90.3)	1998 (93.0)
Yes	167 (7.2)	16 (9.7)	151 (7.0)
Hearing impairment (using hearing aids) (n, %)			
No	2280 (98.5)	159 (96.4)	2121 (98.7)
Yes	34 (1.5)	6 (3.6)	28 (1.3)
Alcohol consumption (n, %)			
Non-drinker	1918 (83.1)	130 (78.8)	1788 (83.4)
1–3 times a week	159 (6.9)	11 (6.7)	148 (6.9)
≥ 4 times a week	232 (10.1)	24 (14.6)	208 (9.7)
Smoking (n, %)			
Non-smoker	1568 (67.8)	95 (57.6)	1473 (68.6)
Ex-smoker	191 (29.9)	61 (37.0)	630 (29.3)
Current smoker	53 (2.3)	9 (5.5)	44 (2.1)
Systolic blood pressure (mean, SD)	121.9 (25.7)	123.2 (19.0)	121.8 (26.2)
Diastolic blood pressure (mean, SD)	76.2 (16.8)	76.1 (14.3)	76.2 (17.0)
Pulse rate (mean, SD)	91.2 (17.3)	94.8 (17.4)	90.9 (17.3)
Respiratory rate (mean, SD)	19.1 (2.7)	19.4 (2.5)	19.1 (2.8)
Body temperature (mean, SD)	36.9 (0.5)	36.9 (0.5)	36.9 (0.6)
History of delirium (n, %)	69 (3.0)	31 (18.8)	38 (1.8)
History of cardiovascular disease (n, %)	892 (38.6)	78 (47.3)	814 (37.9)
History of diabetes mellitus (n, %)	530 (22.9)	52 (31.5)	478 (22.2)
History of respiratory diseases (n, %)	206 (8.9)	19 (11.5)	187 (8.7)
History of liver diseases (n, %)	147 (6.4)	12 (7.3)	135 (6.3)
History of mental illness (n, %)	158 (6.8)	21 (12.7)	137 (6.4)
History of head injury (n, %)	163 (7.0)	17 (10.3)	146 (6.8)
Hemoglobin (mean, SD)	10.6 (2.9)	10.3 (2.2)	10.6 (2.9)
Hematocrit (mean, SD)	31.8 (9.3)	31.0 (6.2)	31.9 (9.5)
White blood cell count (mean, SD)	9.4 (8.8)	10.9 (8.1)	9.3 (8.8)
Platelets (mean, SD)	236.3 (137.2)	235.1 (171.0)	236.4 (134.3)
Aspartate transaminase (mean, SD)	69.3 (216.3)	45.3 (49.4)	71.2 (224.0)
Alanine transaminase (mean, SD)	46.8 (149.7)	28.8 (33.1)	48.2 (155.0)
Blood urea nitrogen (mean, SD)	21.6 (16.2)	25.6 (16.9)	21.2 (16.1)
Cr (mean, SD)	1.0 (1.6)	1.0 (0.7)	1.0 (1.7)
eGFR (mean, SD)	88.7 (31.4)	80.1 (29.9)	89.4 (31.4)
Glucose (mean, SD)	133.62 (58.5)	146.6 (68.8)	132.6 (57.5)
Na (mean, SD)	136.1 (22.1)	134.4 (6.1)	136.2 (22.9)
Cl (mean, SD)	100.0 (7.4)	98.9 (6.9)	100.0 (7.4)
Ca (mean, SD)	9.2 (18.8)	8.9 (1.4)	9.2 (19.5)
K (mean, SD)	4.5 (8.8)	4.3 (0.7)	4.5 (9.1)
Total bilirubin (mean, SD)	1.6 (4.1)	1.3 (2.5)	1.6 (4.2)
Uric acid (mean, SD)	5.0 (7.6)	5.0 (2.6)	5.0 (7.9)
C-reactive protein (mean, SD)	26.6 (49.5)	38.6 (66.5)	25.7 (47.8)

*DBP* diastolic blood pressure, *SD* standard deviation, *SBP* systolic blood pressure.

**Figure 1:**
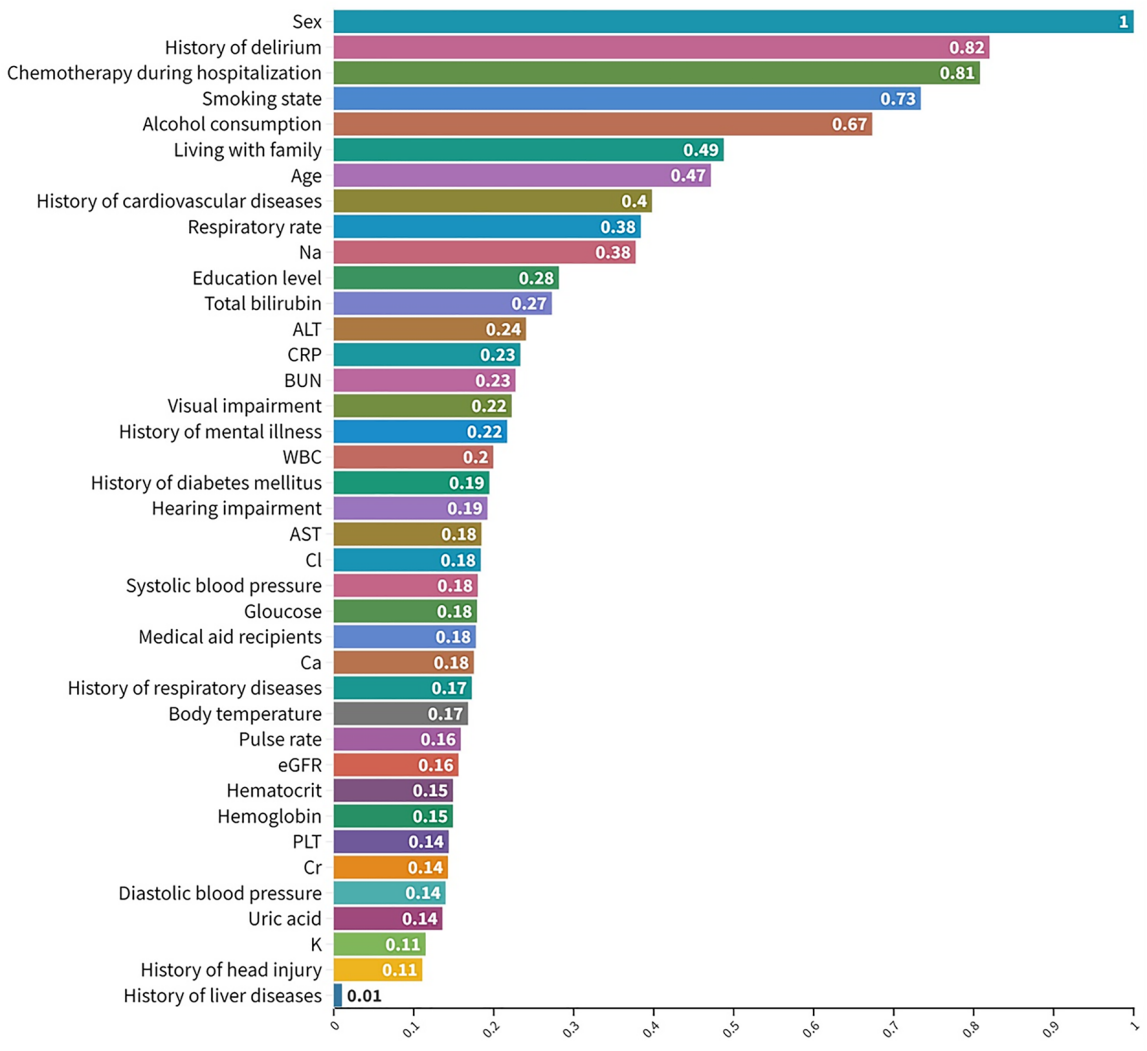
Ranked feature importance values for all 39 features. *WBC* white blood cell count, *PLT* platelets, *AST* aspartate transaminase, *ALT* alanine transaminase, *BUN* blood urea nitrogen.

Table 2 summarizes the fivefold cross validation accuracy comparison of each model and the ensemble machine learning model using the accuracy metrics of sensitivity, specificity, balanced accuracy, and AUROC. In terms of balanced accuracy and AUROC, the three models—RF, XGBoost, and LGB—demonstrated the highest performance compared with the other single models. To further improve classification performance, we adopted an ensemble approach using three single models with higher performance: RF, XGBoost, and LGB. The results revealed that the combination of XGBoost and RF provided the most optimal performance, achieving the following accuracy metrics: 68.83% sensitivity, 70.85% specificity, 69.84% balanced accuracy, and 74.55% AUROC. Subsequently, we performed feature importance analysis using an ensemble model that combines XGBoost and RF. We averaged and normalized the values of feature importance from the two models and ranked each feature. Figure 1 presents the normalized values of ranked feature importance from all 39 features used to predict delirium in patients with advanced cancer. The results indicated that sex (1.00) had the highest importance value and was the primary contributor to predicting delirium, followed by a history of delirium (0.82), chemotherapy during hospitalization (0.81), smoking status (0.73), alcohol consumption (0.67), living with family (0.49), and age (0.47).

**Table 2:** Five-fold cross validation result comparison according to machine learning models.

Model	Training data (n = 1851)			
	Sensitivity, % (SD)	Specificity, % (SD)	Balanced accuracy, % (SD)	AUROC, % (SD)
GB	66.58 (7.96)	64.63 (2.89)	65.60 (3.97)	71.92 (5.87)
LGB	68.03 (9.61)	66.89 (3.54)	67.47 (3.72)	73.60 (5.44)
RF	74.13 (6.48)	64.68 (4.35)	69.41 (1.48)	74.06 (4.12)
SVM	62.05 (11.81)	66.02 (5.11)	64.04 (5.83)	70.42 (5.03)
AdaBoost	58.12 (16.29)	59.39 (13.34)	58.75 (7.56)	64.01 (7.41)
XGBoost	67.21 (16.70)	63.52 (20.43)	65.37 (3.50)	73.81 (5.48)
Logistic regression	57.52 (8.28)	68.18 (1.84)	62.85 (3.71)	69.70 (3.39)
LGB + RF	64.22 (10.28)	71.49 (4.32)	67.85 (4.03)	74.08 (5.13)
**RF + XGB**	**68.83 (8.36)**	**70.85 (4.41)**	**69.84 (4.46)**	**74.55 (4.81)**
LGB + XGB	65.81 (8.22)	70.85 (3.96)	68.33 (4.71)	73.81 (5.13)
LGB + RF + XGB	69.60 (8.18)	65.15 (3.95)	67.38 (4.57)	73.64 (5.14)

*GB* gradient Boosting, *SVM* support vector machine, *AdaBoost* adaptive boosting, *XGBoost* extreme gradient boosting, *RF* random forest.

The combination of XGBoost and RF provided the most optimal performance, as indicated in bold.

We validated the performance of the machine learning models using an isolated testing dataset. Table 3 summarizes the delirium prediction results of the test dataset. The results also showed that the combination of XGBoost and RF provided the most optimal performance with the following accuracy metrics: 75.76% sensitivity, 52.63% specificity, 64.19% balanced accuracy, and 73.11% AUROC. Compared with the fivefold cross validation results, the accuracy metrics of balanced accuracy and AUROC were similar to the testing data results, indicating minimal overfitting or underfitting in the model.

**Table 3:** Delirium prediction results from the testing dataset.

Model	Testing data (n = 463)			
	Sensitivity, % (SD)	Specificity, % (SD)	Balanced accuracy, % (SD)	AUROC, % (SD)
GBM	72.73	55.81	64.27	70.25
Light GBM	75.76	53.72	64.74	71.59
RF	72.73	55.35	64.04	71.47
SVM	66.67	59.53	63.10	66.70
AdaBoost	72.73	63.26	67.99	71.22
XGBoost	51.52	72.09	61.80	68.94
Logistic regression	66.67	63.95	65.31	67.17
LGB + RF	72.73	58.84	65.78	71.71
**RF + XGB**	**75.76**	**52.63**	**64.19**	**73.11**
LGB + XGB	75.76	51.16	63.46	73.09
LGB + RF + XGB	81.82	45.12	63.47	73.06

*GB* Gradient Boosting, *SVM* support vector machine, *AdaBoost* adaptive boosting, *XGBoost* extreme Gradient Boosting, *RF* random forest.

The combination of XGBoost and RF provided the most optimal performance, as indicated in bold.

**Figure 2:**
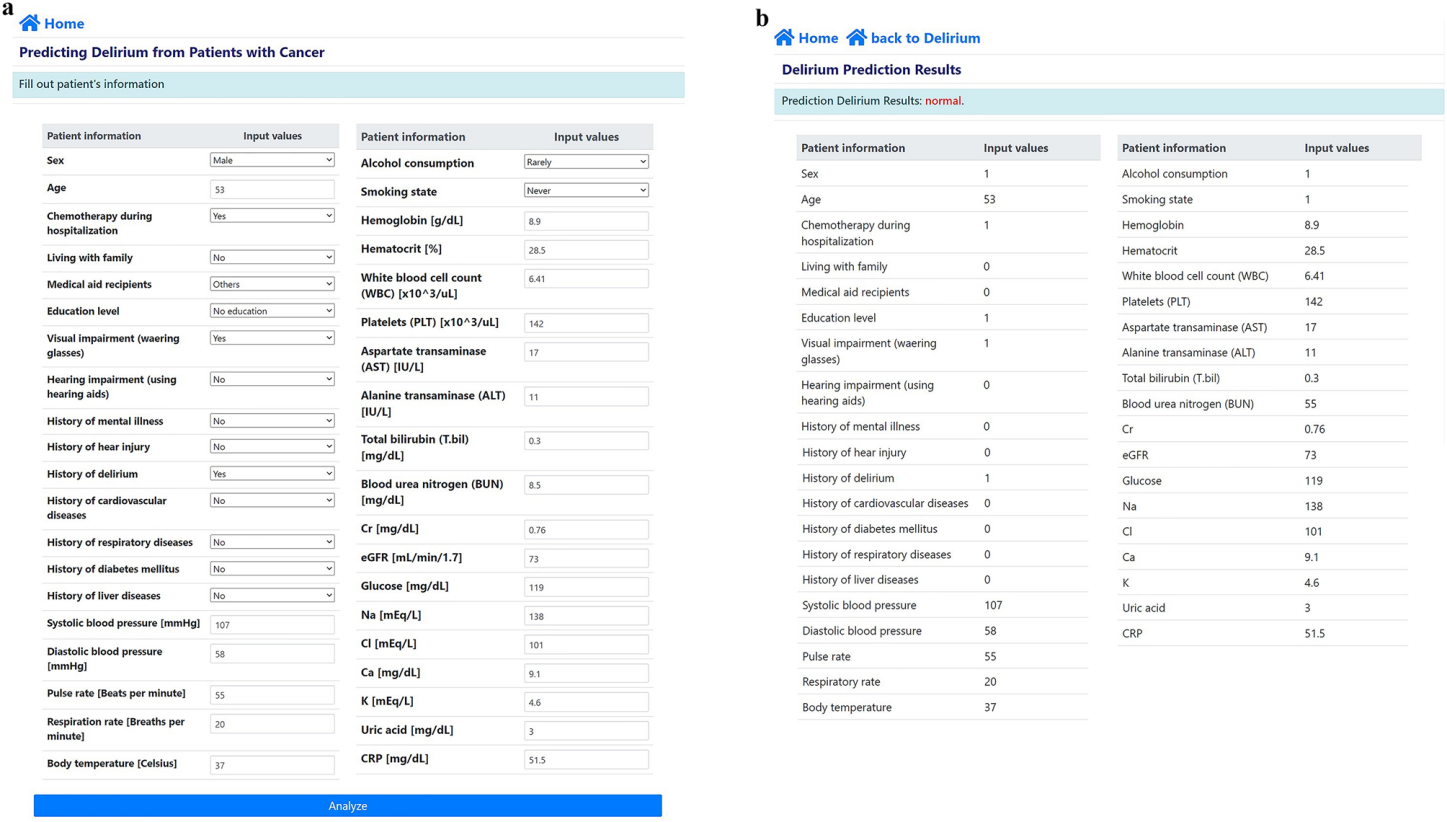
Deployed web application predicting delirium: (**a**) user input, (**b**) prediction results with delirium probability in patients with advanced cancer.

Furthermore, we deployed our artificial intelligence (AI) on a public website (http://ai-wm.khu.ac.kr/Delirium/) to allow public access to the delirium prediction results in patients with advanced cancer. Figure 2 displays the website of the deployed AI model. Figure 2a illustrates the user web interface for entering information, where users inputs 39-feature data such as sex, age, chemotherapy during hospitalization, living with family, medical aid recipients, and education levels. Upon entering the information into the web application, users can immediately obtain the delirium prediction results, as shown in Fig. 2b. The prediction results include the probability of mortality.

## Discussion

### Key findings

The results suggest that machine learning models can predict delirium in patients with advanced cancer admitted to the APCU with relatively high accuracy. The combination model of XGBoost and RF demonstrated the best performance for predicting delirium in these patients, achieving a balanced accuracy of 69.84% and an AUROC of 74.55%. This performance was validated through both k-fold cross-validation and testing on an isolated dataset. Notably, sex emerged as the most critical feature for predicting delirium in patients with advanced cancer, followed by a history of delirium, chemotherapy during hospitalization, smoking status, alcohol consumption, living with family, and advanced age. To the best of our knowledge, this study represents the first attempt to use the machine learning model to predict delirium in South Korean patients with advanced cancer. These findings underscore the importance of delirium screening in APCU-admitted patients with advanced cancer and contribute to identifying the most significant risk factors for this patient group.

### Comparison of previous studies

Our results, particularly in the combination model of XGBoost and RF, corroborate previously reported risk factors associated with delirium. Earlier research indicated that advanced age, a history of delirium, smoking status, alcohol consumption, and sex were associated with delirium in patients with advanced cancer admitted to the APCU^31–34^. Male sex was identified as a significant risk factor for neuropsychiatric disorders, potentially due to the protective role of estrogen in individuals with potential cognitive impairments^35,36^. Males may exhibit more pronounced neuropsychiatric disorders under acute stress, driven by different corticotropin-releasing factor signaling pathways compared with females^37^. Consistent with prior studies, our findings highlight old age as a significant risk factor for delirium in patients with advanced cancer^38–40^, with possible contributing factors being atherosclerosis and malnutrition common in older patients^40–42^. The association of cigarette smoking with delirium is attributed to nicotine withdrawal during hospitalization^1^. Smokers have been noted to display more severe agitation, characteristic of hyperactive delirium^43^. Changes in various neurotransmitter systems, including dopamine, opioids, and cholinergic systems, have been implicated in shared hyperactive delirium^44^. The relationship between chemotherapeutic agents and delirium remains controversial and inconsistent, as reported in single case reports or studies with small populations. Previous studies have suggested that patients who undergo multiple chemotherapy regimens could experience delirium, which may occur in approximately one in 11 adults receiving chemotherapy^45,46^. Chemotherapeutic agents may penetrate the blood–brain barrier, potentially serving as a risk factor for delirium^47,48^. Similar to our study, a previous study was conducted to predict delirium in patients with advanced cancer receiving pharmacological intervention through a visually interpretable prediction model^9^. This study has the advantage of being easy to use with small number of variables, but it is dependent on Delirium Rating Scale Revised-98 and has a limitation in predicting delirium within three days. On the other hand, our study provided a web application with public access with a machine learning model, and could serve as a medical aid for healthcare providers to monitor the delirium in the patients with advanced cancer.

### Strengths and limitations

The primary strength of this study lies in the relatively high accuracy of the machine learning model for detecting delirium in patients with advanced cancer, as validated by testing datasets. Consistently high AUC values in both the training and testing datasets indicate that the combination model of XGBoost and RF is capable of predicting delirium in patients with advanced cancer. Important predictors of delirium include sex, history of delirium, chemotherapy during hospitalization, smoking status, alcohol consumption, living with family, and advanced age. The dataset was collected from four academic cancer centers, involving oncology-trained physicians and healthcare providers, providing a comprehensive view of risk factors associated with delirium in patients with advanced cancer and potentially aiding in the development of effective preventive interventions.

However, this study had several limitations. Firstly, he datasets were collected from patients admitted to four hospitals and were heterogeneous, potentially limiting the generalizability of the model to the general population. Secondly, delirium assessment tools, diagnostic criteria, observation frequency, and timeframes may differ from those used in clinical trials. Thirdly, machine learning models often benefit from larger datasets, but the sample size of this study was limited. Fourthly, our proposed machine learning model underperformed compared to previous studies predicting delirium across varying patient conditions^49,50^. Given the limitations of our registry construction project, we did not collect data at various time points. Additional research may be necessary to address this gap. Fifthly, dataset of this study lacks information pertaining to delirium-related medications or disease history. However, we have initiated the establishment of a new prospective cohort to supplement the inadequate input data values. Consequently, we plan to conduct further research to develop more sophisticated machine learning modeling through subsequent studies. Finally, due to the retrospective design of our registry for patients with advanced cancer, it was not feasible to distinguish between different types of delirium (hyperactivity, hypoactivity, and mixed type). We are fully aware of this limitation, and currently, in our newly established prospective cohort, we are making efforts to differentiate between them. To apply machine learning models and achieve external validation, a larger sample size dataset is required. Lastly, an imbalance in the number of patients in each group may limit the performance of the models^51,52^.

### Clinical and policy implications

To the best of our knowledge, this study represents the first creation of a machine learning model for predicting delirium in patients with advanced cancer admitted to the APCU. The use of this machine learning model for delirium prediction in APCU-admitted patients with advanced cancer can significantly improve patient quality of life and reduce physician workload. Especially for Korean healthcare providers with less educational experience in delirium^53^, the machine learning-based delirium prediction model of patients with advanced cancer could be part of a medical aid. Delirium episodes are particularly common in patients with advanced cancer in the APCU, with prevalence increasing as the terminal phase of the illness approaches. However, delirium in these patients has been inadequately identified and managed. Our model has the potential to profoundly impact risk assessment, early detection, and effective interventions for delirium in patients with advanced cancer.

## Conclusion

Using a large-scale multicenter patient-based registry cohort, we have successfully developed the machine learning prediction model for delirium in South Korean patients with advanced cancer. Our study revealed that the combination of XGBoost and RF delivered the most optimal performance, a conclusion validated by the results of both k-fold cross-validation and the isolated testing dataset. Additionally, we identified sex was the primary predictor of delirium, followed by history of delirium, chemotherapy, smoking status, alcohol consumption, and living with family. Furthermore, we have made our AI accessible to the public through a dedicated website (http://ai-wm.khu.ac.kr/Delirium/) to provide delirium prediction results for patients with advanced cancer. Although external validation using prospectively collected data may be necessary to further refine and validate the model, we have implemented a web application to gather additional data. Notably, the application does not store any user-entered information at present. However, we have plans to securely store the user-entered information with their consent, facilitating a real-time learning process to enhance the machine learning model.

### Supplementary Information

Supplementary Table S1.

## Data Availability

The datasets generated and/or analysed during the current study are not publicly available to Korean medical laws prohibiting the commercial utilization of medical data, its use is restricted for commercial purposes. However the datasets are available from the corresponding author on reasonable academic request.

## References

[1] Fong TG, Tulebaev SR, Inouye SK (2009). Delirium in elderly adults: Diagnosis, prevention and treatment. Nat. Rev. Neurol..

[2] Breitbart W, Gibson C, Tremblay A (2002). The delirium experience: Delirium recall and delirium-related distress in hospitalized patients with cancer, their spouses/caregivers, and their nurses. Psychosomatics.

[3] Lee SW (2023). Risk factors for delirium among patients with advanced cancer in palliative care: A multicenter, patient-based registry cohort in South Korea. Eur. Rev. Med. Pharmacol. Sci..

[4] Hosie A, Davidson PM, Agar M, Sanderson CR, Phillips J (2012). Delirium prevalence, incidence, and implications for screening in specialist palliative care inpatient settings: A systematic review. Palliat. Med..

[5] Sands MB, Wee I, Agar M, Vardy JL (2022). The detection of delirium in admitted oncology patients: A scoping review. Eur. Geriatr. Med..

[6] Smith L (2022). Global Burden of Disease study at the World Health Organization: Research methods for the most comprehensive global study of disease and underlying health policies. Life Cycle.

[7] Ekmekyapar T, Ekmekyapar M, Tasci I, Sahin L, Delen LA (2022). Clinical features and predisposing factors of delirium due to COVID-19 pneumonia in intensive care units. Eur. Rev. Med. Pharmacol. Sci..

[8] Brown EG (2017). Predicting inpatient delirium: The AWOL delirium risk-stratification score in clinical practice. Geriatr. Nurs..

[9] Kurisu K (2022). A decision tree prediction model for a short-term outcome of delirium in patients with advanced cancer receiving pharmacological interventions: A secondary analysis of a multicenter and prospective observational study (Phase-R). Palliat. Support. Care.

[10] Zhang Y (2023). Automated machine learning-based model for the prediction of delirium in patients after surgery for degenerative spinal disease. CNS Neurosci. Ther..

[11] Hur S (2021). A machine learning–based algorithm for the prediction of intensive care unit delirium (PRIDE): Retrospective study. JMIR Med. Inform..

[12] Wong A (2018). Development and validation of an electronic health record-based machine learning model to estimate delirium risk in newly hospitalized patients without known cognitive impairment. JAMA Netw. Open.

[13] Jauk S (2020). Risk prediction of delirium in hospitalized patients using machine learning: An implementation and prospective evaluation study. J. Am. Med. Inform. Assoc..

[14] Liu Y, Shen W, Tian Z (2023). Using machine learning algorithms to predict high-risk factors for postoperative delirium in elderly patients. Clin. Interv. Aging.

[15] Yoo SH (2024). Opioid use and subsequent delirium risk in patients with advanced cancer in palliative care: A multicenter registry study. Sci. Rep..

[16] Pagali SR (2021). Predicting delirium risk using an automated mayo delirium prediction tool: Development and validation of a risk-stratification model. Mayo Clin. Proc..

[17] Bhattacharyya A (2022). Delirium prediction in the ICU: Designing a screening tool for preventive interventions. JAMIA Open.

[18] Jang S, Jung KI, Yoo WK, Jung MH, Ohn SH (2016). Risk factors for delirium during acute and subacute stages of various disorders in patients admitted to rehabilitation units. Ann. Rehabil. Med..

[19] Lee SW (2021). Proton pump inhibitors and the risk of severe COVID-19: A post-hoc analysis from the Korean nationwide cohort. Gut.

[20] Kang J (2024). Prenatal opioid exposure and subsequent risk of neuropsychiatric disorders in children: Nationwide birth cohort study in South Korea. BMJ.

[21] Lee JS (2023). Breastfeeding and impact on childhood hospital admissions: A nationwide birth cohort in South Korea. Nat. Commun..

[22] Sepulveda E (2016). Delirium diagnosis defined by cluster analysis of symptoms versus diagnosis by DSM and ICD criteria: Diagnostic accuracy study. BMC Psychiatry.

[23] Kim J (2023). Quantification of identifying cognitive impairment using olfactory-stimulated functional near-infrared spectroscopy with machine learning: A post hoc analysis of a diagnostic trial and validation of an external additional trial. Alzheimers Res. Ther..

[24] Kim MS (2024). Long-term autoimmune inflammatory rheumatic outcomes of COVID-19: A binational cohort study. Ann. Intern. Med..

[25] Lee H (2024). Machine learning-based prediction of suicidality in adolescents with allergic rhinitis: Derivation and validation in 2 independent nationwide cohorts. J. Med. Internet Res..

[26] Kwon R, Lee H, Kim MS, Lee J, Yon DK (2023). Machine learning-based prediction of suicidality in adolescents during the COVID-19 pandemic (2020–2021): Derivation and validation in two independent nationwide cohorts. Asian J. Psychiatry.

[27] Yoo HW (2021). Non-alcoholic fatty liver disease and COVID-19 susceptibility and outcomes: A Korean nationwide cohort. J. Korean Med. Sci..

[28] Shin YH (2021). Autoimmune inflammatory rheumatic diseases and COVID-19 outcomes in South Korea: A nationwide cohort study. Lancet Rheumatol..

[29] Lee SW (2021). Association between mental illness and COVID-19 in South Korea: A post-hoc analysis. Lancet Psychiatry.

[30] Oh J (2024). Incident allergic diseases in post-COVID-19 condition: Multinational cohort studies from South Korea, Japan and the UK. Nat. Commun..

[31] Inouye SK (2000). Prevention of delirium in hospitalized older patients: Risk factors and targeted intervention strategies. Ann. Med..

[32] Seiler A (2021). Delirium is associated with an increased morbidity and in-hospital mortality in cancer patients: Results from a prospective cohort study. Palliat. Support Care.

[33] Ormseth CH (2023). Predisposing and precipitating factors associated with delirium: A systematic review. JAMA Netw. Open.

[34] Neefjes ECW (2017). Identification of patients with cancer with a high risk to develop delirium. Cancer Med..

[35] Wang H (2021). Gender differences and postoperative delirium in adult patients undergoing cardiac valve surgery. Front. Cardiovasc. Med..

[36] Locklear MN, Kritzer MF (2014). Assessment of the effects of sex and sex hormones on spatial cognition in adult rats using the Barnes maze. Horm. Behav..

[37] Locci A, Yan Y, Rodriguez G, Dong H (2021). Sex differences in CRF1, CRF, and CRFBP expression in C57BL/6J mouse brain across the lifespan and in response to acute stress. J. Neurochem..

[38] Lawlor PG, Bush SH (2015). Delirium in patients with cancer: Assessment, impact, mechanisms and management. Nat. Rev. Clin. Oncol..

[39] Ljubisavljevic V, Kelly B (2003). Risk factors for development of delirium among oncology patients. Gen. Hosp. Psychiatry.

[40] Quinlan N (2011). Vulnerability: The crossroads of frailty and delirium. J. Am. Geriatr. Soc..

[41] Inouye SK, Studenski S, Tinetti ME, Kuchel GA (2007). Geriatric syndromes: Clinical, research, and policy implications of a core geriatric concept. J. Am. Geriatr. Soc..

[42] Inouye SK (2006). Delirium in older persons. The N. Engl. J. Med..

[43] Park H, Kim KW, Yoon IY (2016). Smoking cessation and the risk of hyperactive delirium in hospitalized patients: A retrospective study. Can. J. Psychiatry.

[44] Stagno D, Gibson C, Breitbart W (2004). The delirium subtypes: A review of prevalence, phenomenology, pathophysiology, and treatment response. Palliat. Support. Care.

[45] Jung P (2021). Delirium incidence, risk factors, and treatments in older adults receiving chemotherapy: A systematic review and meta-analysis. J. Geriatr. Oncol..

[46] Wu YC (2019). Association of delirium response and safety of pharmacological interventions for the management and prevention of delirium: A network meta-analysis. JAMA Psychiatry.

[47] Zeevi N, Pachter J, McCullough LD, Wolfson L, Kuchel GA (2010). The blood-brain barrier: Geriatric relevance of a critical brain-body interface. J. Am. Geriatr. Soc..

[48] Matsuoka H, Yoshiuchi K, Koyama A, Otsuka M, Nakagawa K (2014). Chemotherapeutic drugs that penetrate the blood–brain barrier affect the development of hyperactive delirium in cancer patients. Palliat. Support. Care.

[49] Nagata C (2023). Development of postoperative delirium prediction models in patients undergoing cardiovascular surgery using machine learning algorithms. Sci. Rep..

[50] Gong KD (2023). Predicting intensive care delirium with machine learning: Model development and external validation. Anesthesiology.

[51] Zhang Z, Pan L, Deng H, Ni H, Xu X (2014). Prediction of delirium in critically ill patients with elevated C-reactive protein. J. Crit. Care.

[52] McGrane S (2011). Procalcitonin and C-reactive protein levels at admission as predictors of duration of acute brain dysfunction in critically ill patients. Crit. Care.

[53] Kim HY, Kim SO (2024). The effect of nurses’ knowledge and self-confidence on delirium nursing performance of nurses in an integrated nursing and caring services ward: A cross-sectional descriptive study. J. Korean Gerontol. Nurs..

